# Supplementary material for: Strong-coupling of WSe2 in ultra-compact plasmonic nanocavities at room temperature
Source: Nat Commun. 2017 Nov 3;8:1296. doi: 10.1038/s41467-017-01398-3 (PMC5670138; doi:10.1038/s41467-017-01398-3)
Supplement: Supplementary file 1 — Supplementary Information [file 41467_2017_1398_MOESM1_ESM.pdf]

Supplementary Figures:

Supplementary Note 1: Tuning the plasmon resonance in the weak coupling regime

Using similar light-induced re-sculpting of a 65 nm Ag nano-cube on mirror (NCoM), we continuously tune the red-shifting plasmon resonance (Supplementary Fig. 1). As well as the dark-field scattering, the light emission originating from both plasmon and exciton resonances is tracked[1], while the plasmon mode shifts across the visible and near-IR spectral regions. A single layer of MoS_2 (0.7 nm) is used as a spacer, and only the weak coupling regime is observed as the plasmon crosses the A-exciton at 664 nm. At resonance, a relative increase in exciton emission of typically 5-fold is seen.

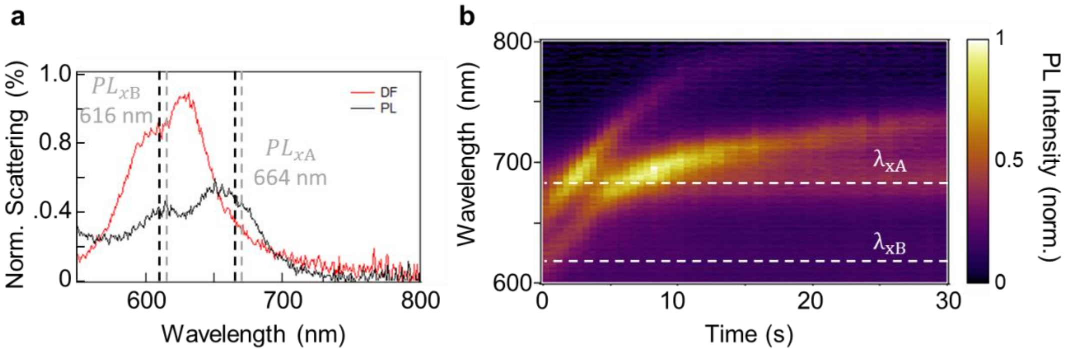

Supplementary Figure 1. Tuning the plasmon resonance of a NCoM (65 nm Ag cubes on single layer MoS_2 on Ag) with a 448nm laser. Comparison of **(a)** dark-field scattering and **(b)** light emission, which show the same resonance peaks.

For larger nanoparticles such as cubes [2] with a cube side (75nm) which supports modes with opposite phase either side of the facet centre but has a side greater than the exction d_c, only weak coupling is allowed to the exciton through s_{11}.

Supplementary Note 2: Reflectivity measurements of excitonic transitions in WSe_2 Flake

Room temperature reflectivity of a 12 multilayer WSe_2 flake on top of a 100 nm Au layer are taken in bright-field illumination with a custom-modified dark-field microscope using a 20x objective (Supplementary Fig. 2a) and simulated (Supplementary Fig. 2b). The two exciton dips corresponding to the A- and B-excitons are marked with orange dashed lines. These reflectivity measurements are recorded at positions remote from the nanoparticles seen in the bright-field image in Supplementary Fig. 2c.

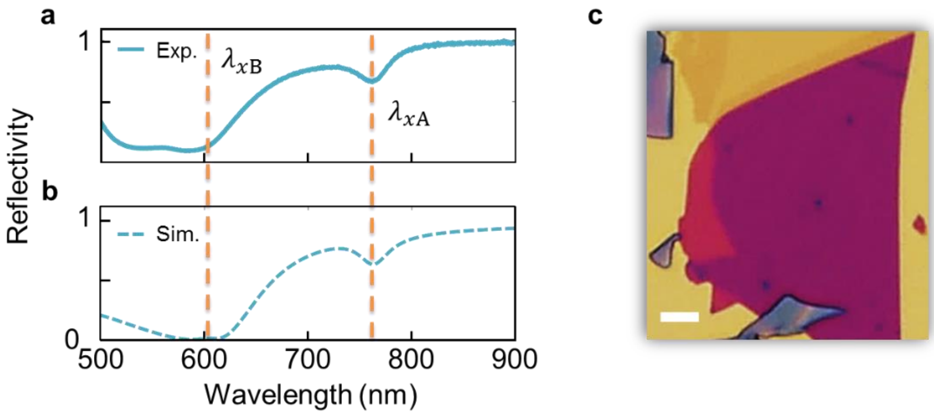

Supplementary Figure 2. Reflectivity of multilayer WSe2 flake. (**a**),(**b**) Experimental (solid) and simulated (dashed) Reflectivity of multilayer WSe_2 flake on Au substrate, recorded away from NPs**,** A- and B- exciton positions indicated (orange dashed lines). **(c)** Bright-field image of the WSe_2 flake on Au. Scale bar (white) corresponds to 10 $\mu\mathrm{m}$.

Supplementary Note 3: Origin of direct and indirect exciton-transitions in single and multilayer WSe_2

TDMs such as WSe_2 change from direct (Supplementary Fig. 3a) to indirect band gap (Supplementary Fig. 3b) when transitioning from single layer to multilayer.[3,4] The direct band gap transition in a monolayer takes place at the degenerate K, K' points at the edge of the Brillouin zone (orange arrow). With increasing number of layers, the band structure changes. The blue-shifted valence band at the Γ-point results in additional competing indirect transitions. Intervalley scattering enables transitions across $\Lambda - \Gamma$ and phonon-assisted transitions across $\mathrm{K} - \Gamma$. However, these processes emit light at different photon energies[4]. While the $\mathrm{K} - \mathrm{K}$ transition has an energy difference of 1.63 eV, the Γ-K transition energy is red-shifted to 1.4 eV. Tuning the plasmon mode into resonance with the direct A-exciton therefore enhances $\mathrm{K} - \mathrm{K}$ recombination rates, rebalancing the branching ratios. The large Purcell enhancement in the plasmonic cavity decreases the A exciton lifetime, which emit before carriers can scatter into the Γ, Λ valleys.

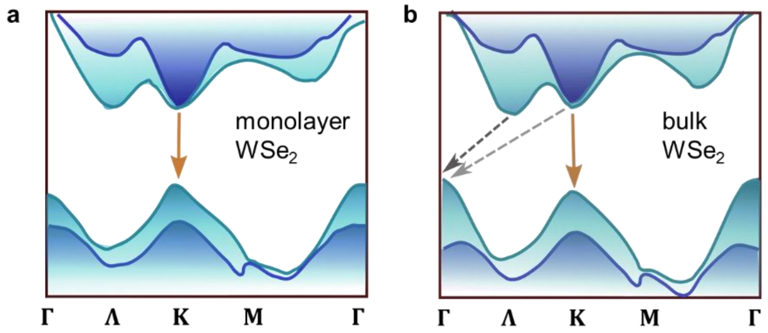

Supplementary Figure 3. Schematic band structures of WSe_2 at room temperature. (**a**) Single monolayer WSe_2 with direct-band gap at the K point. (**b**) Bulk WSe_2 showing the competing relaxation processes. Direct recombination (orange), phonon-assisted inter-valley scattering ($\Lambda - \Gamma$) and phonon-assisted relaxation ($\mathrm{K} - \Gamma$).

Supplementary Note 4: Surface-enhanced Raman scattering (SERS)

The Raman scattering of the 12 multilayer WSe_2 flake on the Au surface (Supplementary Fig. 4, blue) is compared to its SERS in the gap of a NPoM (orange). While the main features of the E_{2g}^1- and A_{1g} modes are well reproduced in both curves and coincide with literature[5], a drastic enhancement of the Raman modes as well as the SERS background is seen in the case of the NPoM, due to the enhanced optical fields. Estimating the size of the localised spot and the laser focus, suggests an actual local enhancement of 5000, as expected from the field enhancement in the main text.

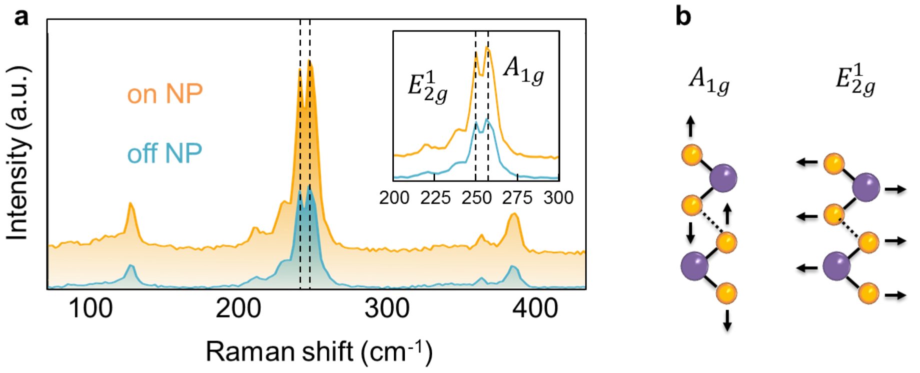

Supplementary Figure 4. Surface-enhanced Raman scattering (SERS). (a) The WSe_2 is clearly identified within the plasmonic gap from SERS of the assembled NPoM. **(b)** Characteristic E_{2g}^1- and A_{1g}-modes are seen in **(a)** at 250 and 257 cm^{-1} respectively. Pump laser at 633 nm, focussed to spot size of 1.2 μm at power of 24 μW.

Supplementary Note 5: Laser induced tuning of 12 layer WSe_2 flake in plasmonic cavity shows strong-coupling signature

The plasmon modes of a 100 nm facetted Au nanoparticle on top of an Au substrate with 12 layers of WSe_2 as spacer is tuned. By irradiating the nanoparticle with 448 nm CW laser light, adatom drifts towards the bottom facet of the nanoparticle lead to a red shifts of the plasmonic gap modes[6]. When tuned close to the A-exciton transition, a sharp dip is seen leading to a splitting in the plasmon resonance. Further tuning leads to the characteristic change in the ratios of the amplitudes of the split modes.

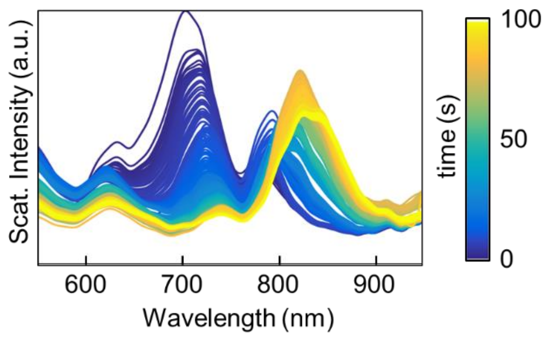

Supplementary Figure 5. Signature of strong coupling in an individual NPoM. Normalized dark-field scattering spectra while red-shifting the plasmon resonance using weak blue-laser irradiation. Colour from blue to yellow with increasing irradiation time.

Supplementary Note 6: Strong-coupling signature for different individual plasmonic cavities

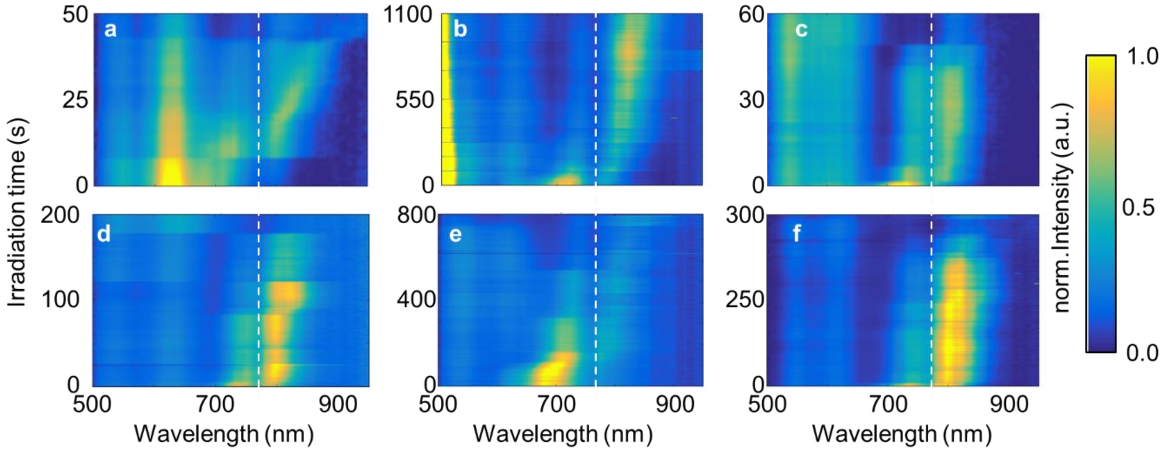

Supplementary Figure 6. Signature of strong coupling in individual NPoM constructs. (**a-f**) Tuning the plasmon mode of different NPoMs with a 448 nm laser, red-shifts the plasmon resonance and always leads to an anti-crossing around the A-exciton resonance. This behaviour is reproduced in all the different individual NPoMs. White dotted line corresponds to A-exciton position.

The tuning of the plasmon resonance is reproducible for many different NPoM constructs. The initial mode positions vary due to variations in size and faceting of each nanoparticle. As shown before, nanoparticles are naturally highly facetted and variations in their shape can be seen even for spherical particles[7]. The coupled mode is highly sensitive to changes in the gap morphology as well as to variations in shape determining the initial mode positions. Regardless of the initial mode position, a monotonic red-shift of the coupled mode is seen with increasing radiation time, while the transverse and the quadrupolar modes remain unaffected.

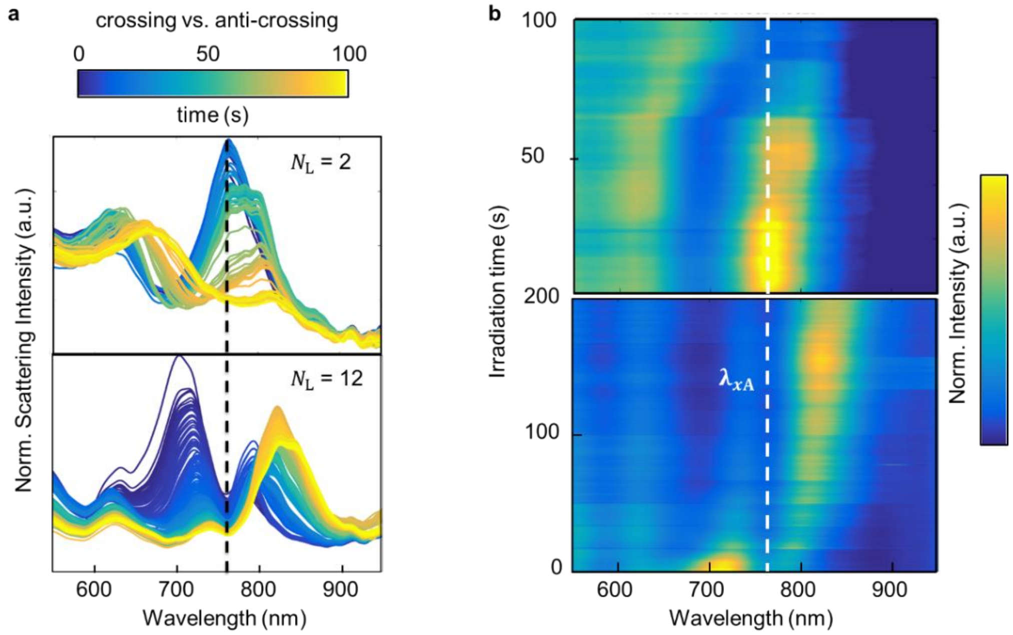

Supplementary Figure 7. Crossing versus anti-crossing. (a) Individual normalized Darkfield scattering spectra with elapsing time. **(b)** dark-field scattering maps showing the time evolution of

the peak intensities colour coded. Upper row shows a crossing in the Dark-field spectra and corresponding dark-field map of the plasmon resonance and the exciton absorption line while laser induced tuning. In contrast in the lower row for the case of 12 Layers a clear anti-crossing is displayed close to the A-exciton.

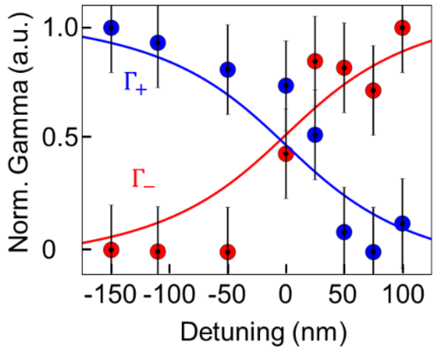

Supplementary Figure 8. Comparison of two coupled Oscillator model to extracted experimental data. The FWHM was extracted by fitting 2 Lorenzians to the experimental data. Error bars stem from standard error of fitted linewidth.

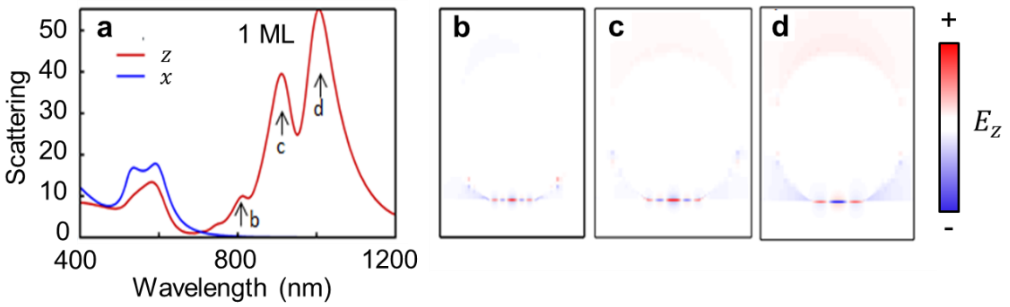

Supplementary Figure 9. FDTD simulations of scattering spectra for NPoM with gap of 1ML WSe_2. (**a**) Scattering spectra for x, z polarisations showing only z resonances around exciton wavelength. (**b-d**) Field distributions of E_z at the three near-IR modes as marked in (a). Oscillatory features are wider than the exciton decoherence size, resulting in minimal exciton oscillator strength into these modes.

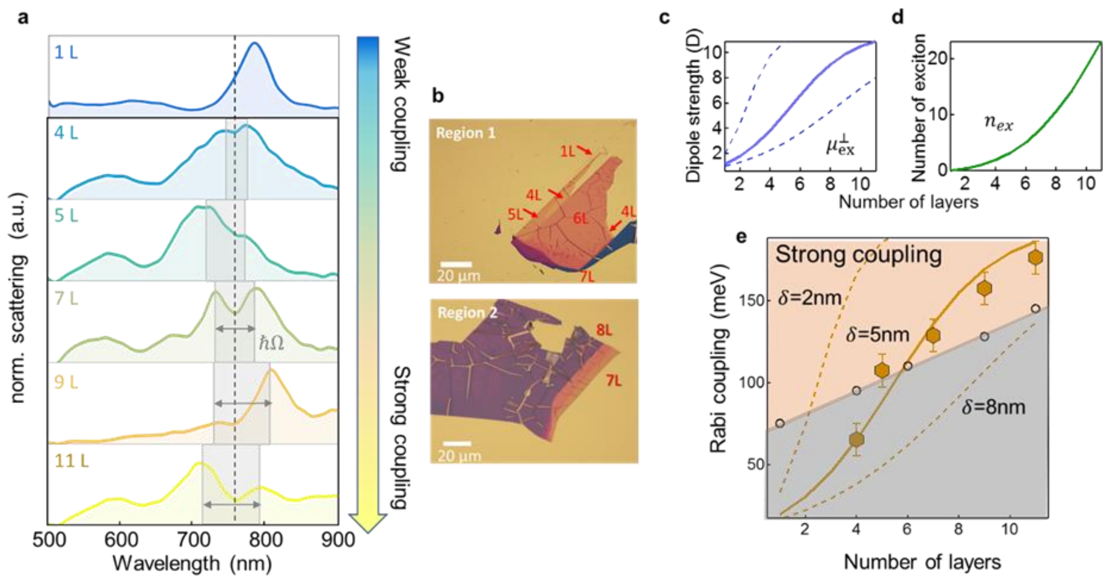

Supplementary Figure 10. **Layer dependent transition from weak coupling to strong coupling regime.** (**a**) Measured dark-field scattering spectra for NPoMs with different N_{L} showing clear splitting for $N_{\mathrm{L}} \geq 7$. (**b**) Bright field images of exfoliated WSe_2 with different layers N_{L}, confirmed by AFM. (**c**) Analytically calculated z-dipole strength (in Debye) of exciton with increasing N_{L} for different interlayer coupling lengths δ (see main text, solid line 5nm, dashed 2nm and 8nm), maximum dipole contribution is 20% total exciton strength. (**d**) Calculated number of excitons within the mode volume. (**e**) Using the dipole in (c) fits the experimentally obtained Rabi coupling strength. Grey points are from measured plasmon linewidths, where NPoM size is changed to keep the exciton on resonance, giving strong coupling regime as orange shaded region. Error bars from standard error of fitted Rabi splitting.

Supplementary Note 7: Simulation parameters

In order to tune the plasmon resonance position to the A-exciton in simulations, the NP was adjusted in size and shape accordingly, where the facet size is estimated from previous work[7]:

1 Layer: 40 nm NP with a facet of 25 nm
3 Layers: 60 nm NP with a facet of 25 nm
6 Layers: 80 nm NP with a facet of 30 nm
8 Layers: 100 nm NP with a facet of 40 nm

Supplementary Tables:

Supplementary Table 1: References for comparing nonlinear figures of merit

Source	T= 300	Emitter/ exciton material	Cavity type	λ (nm)	n_g	Ω_R (meV)	$Q = \frac{\lambda}{\Delta\lambda}$	V_m [x10^3] μm^3	$\frac{Q}{V_m} * V_0$
here	✓	WSe_2 multilayer	LP NPoM	761	2.9	140	25	0.02	86500
[8]	X	$MoSe_2$ + hBN	open DC	747	4.5	20/29	6375	15.8	20964
[9]	X	GaSe+ ML $MoSe_2$	DC	613	5	-	7400	1600	133
[10]	77K	MoS_2 ML	Ag SLP	630	4.5	43-116	41	7.8	166
[11]	✓	GaAs QWs	TC	854	3.9	10	73	20	283
[12]	✓	MoS_2	DC	662	4.5	46	4806	135	1288
[13]	✓	WS_2 monolayer	DC	619	3.9	70	790	15.7	1473
[14]	✓	WSe_2 monolayer	TC	751	4.2	23.5	110	0.40	14572
[15]	thy	$MoSe_2$	DC,TC	785	5	20	200	8.7	1391
[16]	✓	WSe_2 (260nm)	WG	790	4.2		14	2.3	371
		WS_2 32nm flake		605	3.9	270/780	61	0.33	5102
		WS_2 Monolayer		615	3.9	101	30	1.7	507
		WS_2 Monolayer		615		60	20	0.31	1871

Supplementary Table 1. Literature overview of strong coupling in thin layers. DC: dielectric cavity, TC: Tamm cavity, LP: localised plasmon, WG: waveguide, SLP: surface lattice plasmon, NPoM: nanoparticle-on-mirror plasmon cavity.

Supplementary References:

1. Mertens, J., Kleemann, M.-E., Chikkaraddy, R., Narang, P. & Baumberg, J. J. How Light Is Emitted by Plasmonic Metals. *Nano Lett.* **17,** 2568–2574 (2017).
2. Chikkaraddy, R. *et al.* How Ultranarrow Gap Symmetries Control Plasmonic Nanocavity Modes: From Cubes to Spheres in the Nanoparticle-on-Mirror. *ACS Photonics* **4,** 469–475 (2017).
3. Zhao, W. *et al.* Origin of indirect optical transitions in few-layer MoS_2, WS_2, and WSe_2. *Nano Lett.* **13,** 5627–5634 (2013).
4. Arora, A. *et al.* Excitonic resonances in thin films of WSe_2 : from monolayer to bulk material. *Nanoscale* **7,** 10421–10429 (2015).
5. Zhao, W. *et al.* Lattice dynamics in mono- and few-layer sheets of WS_2 and WSe_2. *Nanoscale* **5,** 9677 (2013).
6. Mertens, J. *et al.* Tracking optical welding through groove modes in plasmonic nano-cavities. *Nano Lett.* **16,** 5605–5611 (2016).
7. Kleemann, M.-E. *et al.* Revealing Nanostructures through Plasmon Polarimetry. *ACS Nano* **11,** 850–855 (2017).
8. Dufferwiel, S. *et al.* Exciton-polaritons in van der Waals heterostructures embedded in tunable microcavities. *Nat. Commun.* **6,** 8579 (2015).
9. Schwarz, S. *et al.* Two-Dimensional Metal–Chalcogenide Films in Tunable Optical Microcavities. *Nano Lett.* **14,** 7003–7008 (2014).
10. Liu, W. *et al.* Strong Exciton–Plasmon Coupling in MoS2 Coupled with Plasmonic Lattice. *Nano Lett.* **16,** 1262–1269 (2016).
11. Grossmann, C. *et al.* Tuneable polaritonics at room temperature with strongly coupled Tamm plasmon polaritons in metal/air-gap microcavities. *Appl. Phys. Lett.* **98,**

(2011).
12. Liu, X. *et al.* Strong light–matter coupling in two-dimensional atomic crystals. *Nat. Photonics* **9,** 30–34 (2015).
13. Flatten, L. C. *et al.* Room-temperature exciton-polaritons with two-dimensional WS2. *Sci. Rep.* **6,** 1605 (2016).
14. Lundt, N. *et al.* Room-temperature Tamm-plasmon exciton-polaritons with a WSe_2 monolayer. *Nat. Commun.* **7,** 13328 (2016).
15. Lundt, N. *et al.* Monolayered $MoSe_2$: a candidate for room temperature polaritonics. *2D Mater.* **4,** 15006 (2016).
16. Fei, Z. *et al.* Nano-optical imaging of WSe 2 waveguide modes revealing light-exciton interactions. *Phys. Rev. B* **94,** (2016).
